# Modelling the risk of Japanese encephalitis virus in Victoria, Australia, using an expert-systems approach

**DOI:** 10.1186/s12879-023-08741-8

**Published:** 2024-01-08

**Authors:** Mariel Flores Lima, Jacqueline Cotton, Monique Marais, Robert Faggian

**Affiliations:** 1 Centre for Regional and Rural Futures, Faculty of Science, Engineering and Built Environment, Deakin University, Melbourne, VIC Australia; 2 National Centre for Farmer Health, School of Medicine, Deakin University, Hamilton, VIC Australia

**Keywords:** Japanese encephalitis virus, Modelling, Geographic information system, Analytical hierarchy process, Climate change

## Abstract

Predictive models for vector-borne diseases (VBDs) are instrumental to understanding the potential geographic spread of VBDs and therefore serve as useful tools for public health decision-making. However, predicting the emergence of VBDs at the micro-, local, and regional levels presents challenges, as the importance of risk factors can vary spatially and temporally depending on climatic factors and vector and host abundance and preferences. We propose an expert-systems-based approach that uses an analytical hierarchy process (AHP) deployed within a geographic information system (GIS), to predict areas susceptible to the risk of Japanese encephalitis virus (JEV) emergence. This modelling approach produces risk maps, identifying micro-level risk areas with the potential for disease emergence. The results revealed that climatic conditions, especially the minimum temperature and precipitation required for JEV transmission, contributed to high-risk conditions developed during January and March of 2022 in Victora. Compared to historical climate records, the risk of JEV emergence was increased in most parts of the state due to climate. Importantly, the model accurately predicted 7 out of the 8 local government areas that reported JEV-positive cases during the outbreak of 2022 in Victorian piggeries. This underscores the model’s potential as a reliable tool for supporting local risk assessments in the face of evolving climate change.

## Introduction

Vector-borne diseases (VBDs) have been spreading geographically, which is largely attributed to climate change [1–4]. As new areas become suitable for vectors and pathogens, the threat to unexposed populations grows [5]. Japanese encephalitis virus (JEV) is a zoonotic disease transmitted by mosquitoes endemic to Southeast Asia and the Western Pacific [6]. In 2022, Australia experienced a geographic expansion of JEV, spreading across four states and 80 piggeries [7] representing the first time transmission of JEV has occurred beyond the Torres Starit and Cape York in the far northeast of Australia. Climate conditions elsewhere have been associated with the spread of JEV [8–10].

Models using climate change projections provide insights into the potential future range of vectors and associated diseases [11]. By gauging the habitat suitability of vectors in response to climate change, it is possible to predict vector expansion [12]. Identifying regions where the climate might suit pathogens, vectors, and reservoir hosts, can help with advanced preparation and advocacy [11]. Nevertheless, modelling VBDs can be challenging due to the complex interactions between the vector, pathogen, hosts, and environmental conditions [13]. Moreover, vectors can have different requirements in different geographic areas due to localised factors [14–16].

Among predictive models for VBDs, the Environmental Niche Models (ENMs) are popular. They estimate areas suitable for pathogen transmission by mapping the geographic distribution of vectors [17, 18]. A distinct advantage of ENMs is their ability to function without needing data on the interactions between the environment, vectors, hosts, and pathogens [17]. Yet, their efficacy is contingent upon the availability and accuracy of presence/absence records of vectors or pathogens, potentially constraining their results [17].

While software exists for developing an ENM [19], there is a lack of evidence that local and state governments in Victoria, Australia use such tools for their risk assessment [20]. Predominantly, their risk assessments draw on historical data and past incidents, which can limit the prediction of disease emergence [20].

A geographic information system (GIS) can also be useful to VBD modelling efforts. It assimilates diverse environmental variables within a geographically delineated grid of pixels, thereby pinpointing the environmental suitability for a given species and predicting its potential distribution [21]. Modelling the VBD risk with GIS is relevant to target efforts in specific locations [22]. When coupled with multi-criteria decision analysis (MCDA) [23, 24], it creates a spatial multicriteria decision analysis that encompasses geographically specific alternatives and aids spatial decision-making [23]. Applying such an approach to VBD modelling offers actionable insights based on ranking alternatives and sensitivity analysis within targeted locations [23]. Integrating spatiotemporal disease modelling can help to identify and understand variations over time and space while considering environmental and sociodemographic factors [25].

The analytical hierarchy process (AHP) is a decision-making tool that breaks down complex problems into interrelated decisional elements and arranges them into a hierarchical structure [23, 26]. Several applications of the AHP and other MCDA in healthcare were discussed previously as a solid method to use in decision-making in this field [27]. Additionally, geospatial technologies have been previously used by decision-makers to map vulnerable and high-risk locations and to support public health decisions, such as the case of COVID-19 response programs launched by the WHO since 2020 [28].

The combination of GIS and AHP in modelling for VBDs has successfully mapped high-risk areas in a number of previous studies [29–35]. Such a GIS-AHP-based model can support local government efforts in assessing disease emergence risk, especially by identifying areas where the disease may be established due to climatic shifts. Furthermore, this approach can catalyse effective public health policy formulation, medication and vaccination distribution, business intelligence insights, and healthcare infrastructure planning [25]. It does so by illuminating patterns, demarcating high-risk zones, and identifying communities susceptible to infectious diseases [25].

The 2022 outbreak of JEV took an unexpected turn, reaching the southern regions of Australia where the disease’s presence was previously unanticipated [36]. The prediction of VBDs in Australia’s temperate climates remains an area requiring further investigation. Tools designed to predict potential outbreaks – both temporally and spatially—are essential for enhancing preparedness and implementing effective mitigation strategies [37].

Our objective is to develop a GIS-AHP model designed to identify potential JEV risk zones in Victoria, especially considering the implications of climate change. Beyond its immediate application, this model holds promise as a versatile framework to determine the risk of other VBDs and as a decision support tool for local policymakers, public health authorities, land-use planners, and scholars.

## Methods

### Study design

**Fig. 1:**
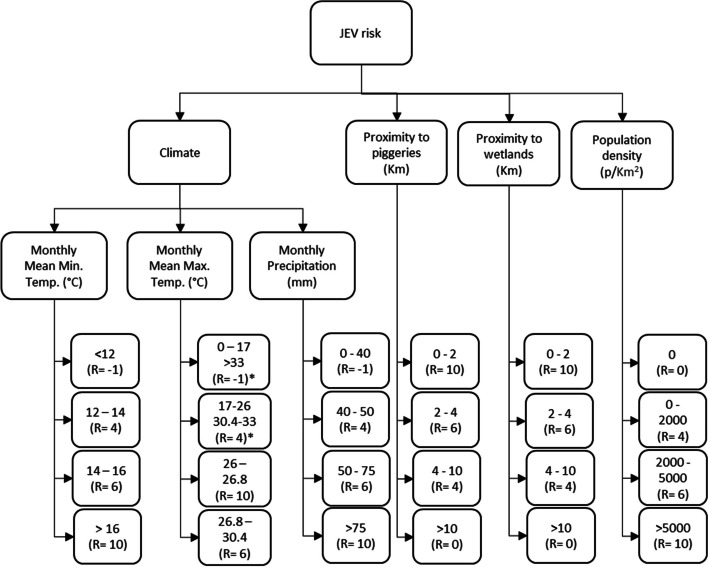
Analytical hierarchy process for the risk of JEV emergence in Victoria. R = rating. * Two ranges of temperature are shown with the same rating

We employed the AHP methodology in conjunction with a GIS to evaluate the potential risk of JEV emergence in Victoria (Fig. 1). The design was structured around three hierarchical levels:

1. Primary Objective: Ascertain the risk of JEV emergence.
2. Criteria Selection: Drawing from the literature, four key criteria were earmarked; climate, proximity to piggeries, proximity to wetlands, and human population density.
3. Detailing Climate Conditions: The third level focused on specific climate conditions pertinent to JEV transmission (Fig. 1).

Within each criterion were identified alternatives characterised by a spectrum of values (Fig. 1). Each value was assigned a rating from -1 to 10, where:

-1: indicates conditions are not met, and the model excludes the corresponding area from consideration.
- 0 – 4: denotes low risk,
- 4 – 6: Signifies medium risk.
- 6 – 10: Represents high risk.

The criteria for maximum and minimum temperatures was set according to a previous study conducted by Mordecai et al. [38]. Since there was no data for the transmission of JEV, the values were derived based on the transmission dynamics of the Murray Valley encephalitis virus (MVEV) by the mosquito *Culex* (*Cx*.) *annulirostris.* MVEV is genetically related to the JEV and is transmitted by a common vector [39]. Temperatures exceeding 33°C or falling below 17°C were assigned a -1 rating, as were minimum temperatures below 12°C.

Precipitation values were set using Victoria’s historical summer season monthly average, approximated at 40 mm [40]. Risk levels based on precipitation were demarcated as follows:

- Low Risk: A rainfall increase of up to 25% above the historical average.
- Medium Risk: A rise in rainfall between 25 and 85% above the historical average.
- High Risk: Rainfall exceeding 85% above the historical average.
-1 (Not Suitable): Monthly averages falling below the 40mm threshold.

Criteria regarding proximity to piggeries and wetlands were identified due to their relevance in the disease emergence process. Pigs and waterbirds act as amplifying hosts, and wetlands provide a suitable habitat for *Cx. annulirostris* [36, 41]. Distances from these focal points were determined in kilometres (km), reflecting the dispersal distance capabilities of the mosquito *Cx. annulirostris*. Specifically, the average flying distance of this species is up to 2 km and a maximum distance of 10 km [41]. Accordingly, the risk values were assigned as:

- 0 – 2 km: Rated 10.
- 2 – 4 km: Rated 6.
- 4 – 10 km: Rated 4.
- Above 10 km: Rated 0.

Population density was the final criterion, with value ranges informed by the Australian Bureau of Statistics [42]. The classifications were as follows:

- No population: Rated 0.
- Up to 2000 people/km^2^ (Low Density): Rated 4.
- 2000 – 5000 people/km^2^ (Medium Density): Rated 6.
- Above 5000 people/km^2^ (High Density): Rated 10.

### Expert panel

We engaged a panel of 12 experts drawn from various fields: epidemiology, mosquito control, disease surveillance, mosquito-borne diseases, and entomology. All participants involved in this study provided a written informed consent.

The role of the expert panel was to ascertain the relative importance of each criterion. To facilitate this, experts undertook a pairwise comparison for each criterion, using a 1 – 9 scale. The scale was defined as follows:

- 1: Both criteria are of equal importance.
- 3: Moderate preference for one criterion over the other.
- 5: Strong preference for one criterion.
- 7: Very strong preference for one criterion.
- 9: One criterion is extremely favoured over the other.

For finer distinctions, intermediate values were also permitted.

Following the evaluations, the scores given by each expert were normalised using a matrix, according to Saaty’s eigenvector procedure [26]. For the evaluations to be deemed consistent, each participant’s consistency ratio (CR) had to fall within the range of 0—0.1 [26].

Having consolidated the weights for each criterion, we then constructed the model. Visualisation and display were facilitated using the Model Builder tool in ArcMap 10.7.1 [43].

### Study area

Victoria, situated in the southeastern region of Australia, spans an area of 227,444 km^2^ [44]. As of 2022, it is home to approximately 6.7 million people, making it the second most populous state in Australia [45].

### Data preparation

All data files underwent reprojection to conform to the GCS_GDA2020 coordinate system. Considering the flight range of the mosquito *Cx. annulirostris*, a buffer zone of 0 to 10 km was established to gauge proximity to both piggeries and wetlands.

### Baseline map

The baseline map for the state of Victoria was obtained from the Australian Statistical Geography Standard (ASGS) in a GDA94 digital boundary shape file [46].

### Climate data

- Historical Data: Monthly average minimum and maximum temperatures, as well as precipitation data from 1970 to 2000, were extracted from the WorldClim website. The data (version 2.1) features a spatial resolution of 30 s (~ 1 km^2^) [47].
- Recent Data: Climate data specific to the period December 2021 to March 2022 for Victoria were acquired from the Australia Bureau of Meteorology (BoM) [48]. This data encompasses monthly maximum and minimum temperatures, in addition to precipitation records from 809 weather stations across Victoria. The climate data was geocoded and interpolated using Kriging to spatially map the data for analysis at 30-s spatial resolution.

### Data sources

1. Wetlands: The location of wetlands was sourced from the Victorian Wetland Inventory [49].
2. Piggeries: The location of piggeries was obtained from the Farm Transparency Project [50]. Only operating pig farms within Victoria were included in this study, based on their GPS locations.
3. Human population density:
  Population data for 1991 was obtained from the Australian Bureau of Statistics [51],
  Data for 2021 was obtained from the Australian Bureau of Statistics [52].

The population density was calculated by dividing the number of people living in a Statistical Areas Level 2 (SA2s) by the size of an SA2 in km^2^.

### Sensitivity analysis

Within the framework of AHP, sensitivity analysis typically revolves around the weights of criteria [23]. The main objective is to determine the impact of weight variations on the final outputs. Should the outputs remain consistent despite these weight fluctuations, it can be inferred that the margin of error in estimating attribute weights is negligible [23].

Our approach to sensitivity analysis involved adjusting the original AHP-calculated weight for each criterion by ± 15% and ± 25%. Meanwhile, other criteria weights were modified proportionally, ensuring the cumulative weight remained one (Table 1).

**Table 1:** Sensitivity analysis

Criteria	0	1	2	3	4	5	6	7	8	9	10	11	12	13	14	15	16
	Original weights	-15%	-25%	15%	25%	-15%	-25%	15%	25%	-15%	-25%	15%	25%	-15%	-25%	15%	25%
Climate	40	34	30	46	50	41	42	39	38	41	42	39	38	40	41	40	39
Proximity to piggeries	27	29	30	25	24	23	20	31	34	28	29	26	25	27	28	27	26
Proximity to wetlands	24	26	27	22	21	25	26	23	22	20	18	28	30	24	25	24	23
Population density	9	11	12	7	6	10	11	8	7	10	11	8	7	8	7	10	11

Sixteen sensitivity analyses were conducted. The highlighted rows indicate the criterion under analysis. The percentages represent the variation being applied to the criterion’s weight under analysis. Other criteria weights are modified proportionally to keep the sum of the criteria’s weights equal to 1

To calculate the model’s output variations under each sensitivity test, the percentage of pixels for each risk level (low, medium, and high) was calculated using the tabulate areas tool from ArcGIS. Furthermore, distinctive maps were generated for each sensitivity analysis.

The sensitivity analysis was conducted in two sets of data for January:

- Using historical climate data.
- Incorporating climate data from 2022.

## Results

Using the GIS-AHP methodology, we constructed a model to identify regions within Victoria at risk of JEV emergence. Resulting risk maps were generated for the period from December to March, integrating both historical climate data and records from the period December 2021 to March 2022.

Table 2 provides an overview of the weights assigned to each criterion as per the expert panel’s consensus, in conjunction with the consistency ratios (CRs).

**Table 2:** Weights for each criterion are determined by the experts’ scores from the pairwise comparison and the AHP

Risk factors	Experts’ weights												Consolidated weight
	A	B	C	D	E	F	G	H	I	J	K	L	
Precipitation	0.814	0.474	0.452	0.760	0.614	0.258	0.481	0.699	0.196	0.674	0.714	0.467	**0.598**
Minimum Temperature	0.072	0.474	0.476	0.144	0.268	0.637	0.114	0.237	0.311	0.101	0.143	0.067	**0.230**
Maximum Temperature	0.114	0.053	0.072	0.096	0.117	0.105	0.405	0.064	0.493	0.226	0.143	0.467	**0.172**
Consistency ratio	0.060	0.000	0.000	0.080	0.080	0.040	0.030	0.100	0.060	0.090	0.000	0.000	
Climate	0.143	0.332	0.400	0.565	0.470	0.335	0.637	0.224	0.256	0.080	0.656	0.357	**0.403**
Proximity to piggeries	0.033	0.046	0.377	0.231	0.080	0.432	0.219	0.538	0.558	0.514	0.129	0.460	**0.272**
Proximity to wetlands	0.532	0.575	0.169	0.160	0.353	0.152	0.097	0.183	0.050	0.363	0.091	0.149	**0.236**
Human population density	0.292	0.046	0.054	0.044	0.098	0.081	0.047	0.054	0.136	0.043	0.124	0.034	**0.089**
Consistency ratio	0.090	0.080	0.030	0.090	0.070	0.080	0.050	0.020	0.070	0.060	0.030	0.090	

Looking into the criteria nested within the climate category:

1. Precipitation emerged as the most important criterion influencing JEV risk emergence in Victoria, accounting for 60% (0.60) of the total weight.
2. Minimum temperature followed at 23% (0.23).
3. Maximum temperature was given a weight of 17% (0.17).

Considering the hierarchy’s second tier of factors affecting JEV emergence risk:

1. Climate was the most significant determinant, with a weight of 40% (0.40).
2. Proximity to piggeries was next with 27% (0.27).
3. Proximity to wetlands weighted 24% (0.24).
4. Human population density was weighted the least, at 9% (0.09).

The distribution highlights the prioritisation by the experts of climate factors, notably precipitation, in the assessment of JEV emergence risk in Victoria. Additionally, the proximity to piggeries and wetlands also holds significant relevance, emphasising the role of vectors and hosts in disease spread.

### Climate suitability

#### Minimum temperature suitability

**Fig. 2:**
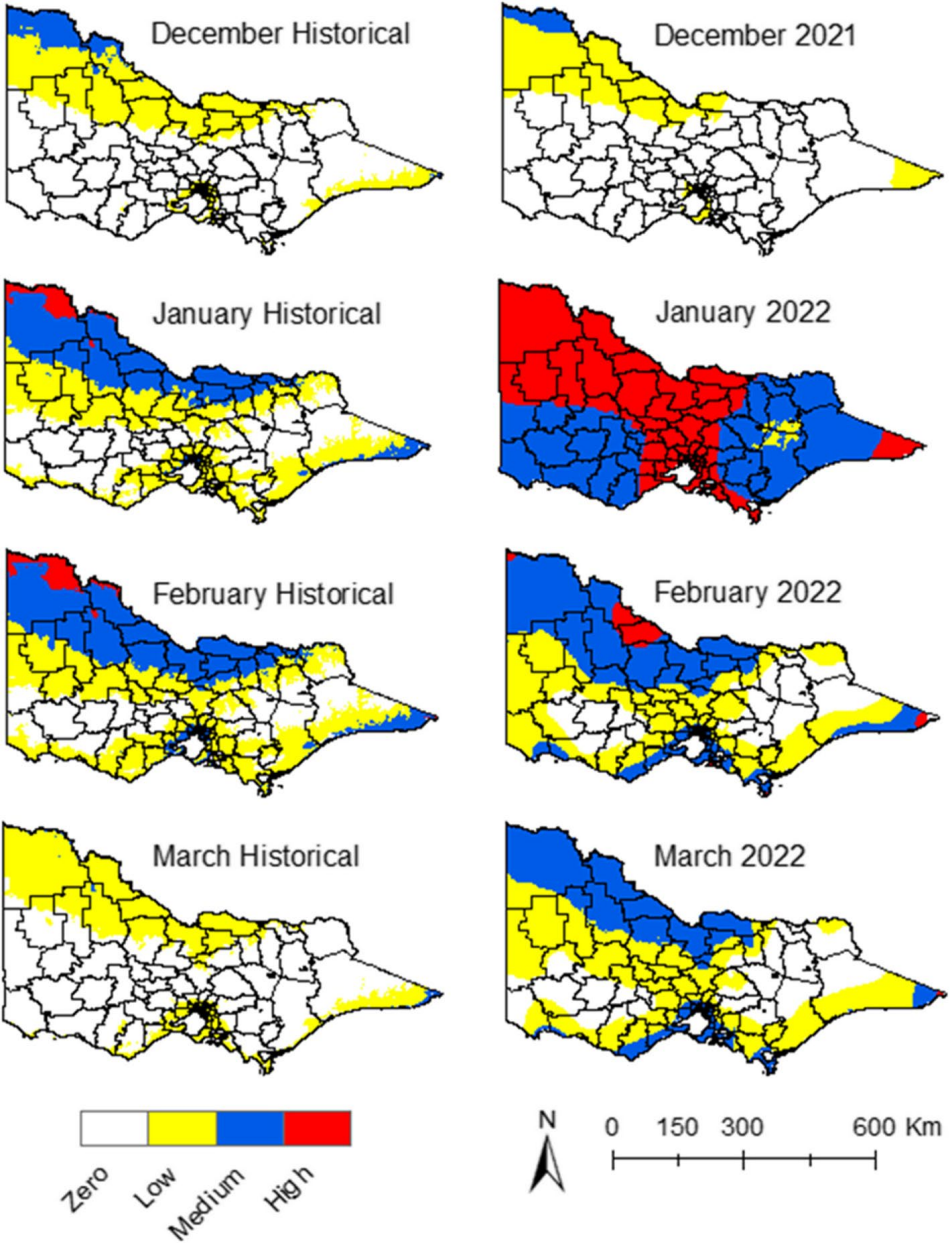
Minimum temperature suitability maps for JEV transmission

The historical data maps illustrate a pattern where, during January and February, the LGAs in the north and northwest of the state were more predisposed to medium and high suitability for JEV transmission. This propensity decreases slightly in December and March (Fig. 2). A notable shift in the patterns can be observed during the summer of 2021–22. Specifically, during January, all LGAs registered increased suitability compared to historical records, with a broad swathe of areas presenting as high and medium suitability (Fig. 2).

#### Maximum temperature suitability

**Fig. 3:**
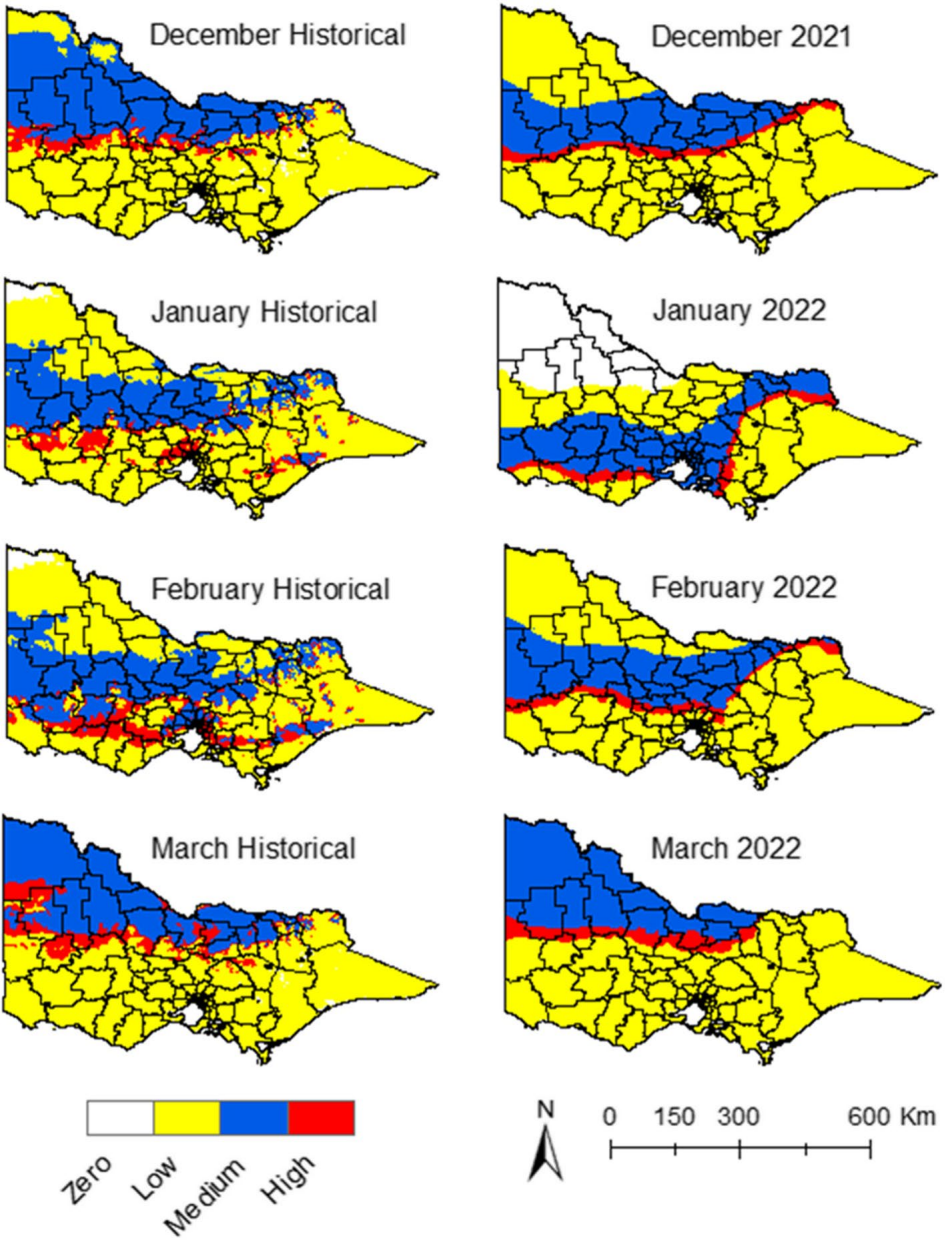
Maximum temperature suitability maps for JEV transmission

In accordance with the historical data, the central belt of the state – during January and February – had areas with medium and high suitability for JEV transmission. This shifted slightly northward in December and March (Fig. 3). However, during January of 2022, the high and medium maximum temperature suitability migrated to the southern and southwest regions. Contrastingly, the northwest corner of the state became too hot to sustain vector populations and thus unsuitable for JEV transmission (Fig. 3).

#### Precipitation suitability

**Fig. 4:**
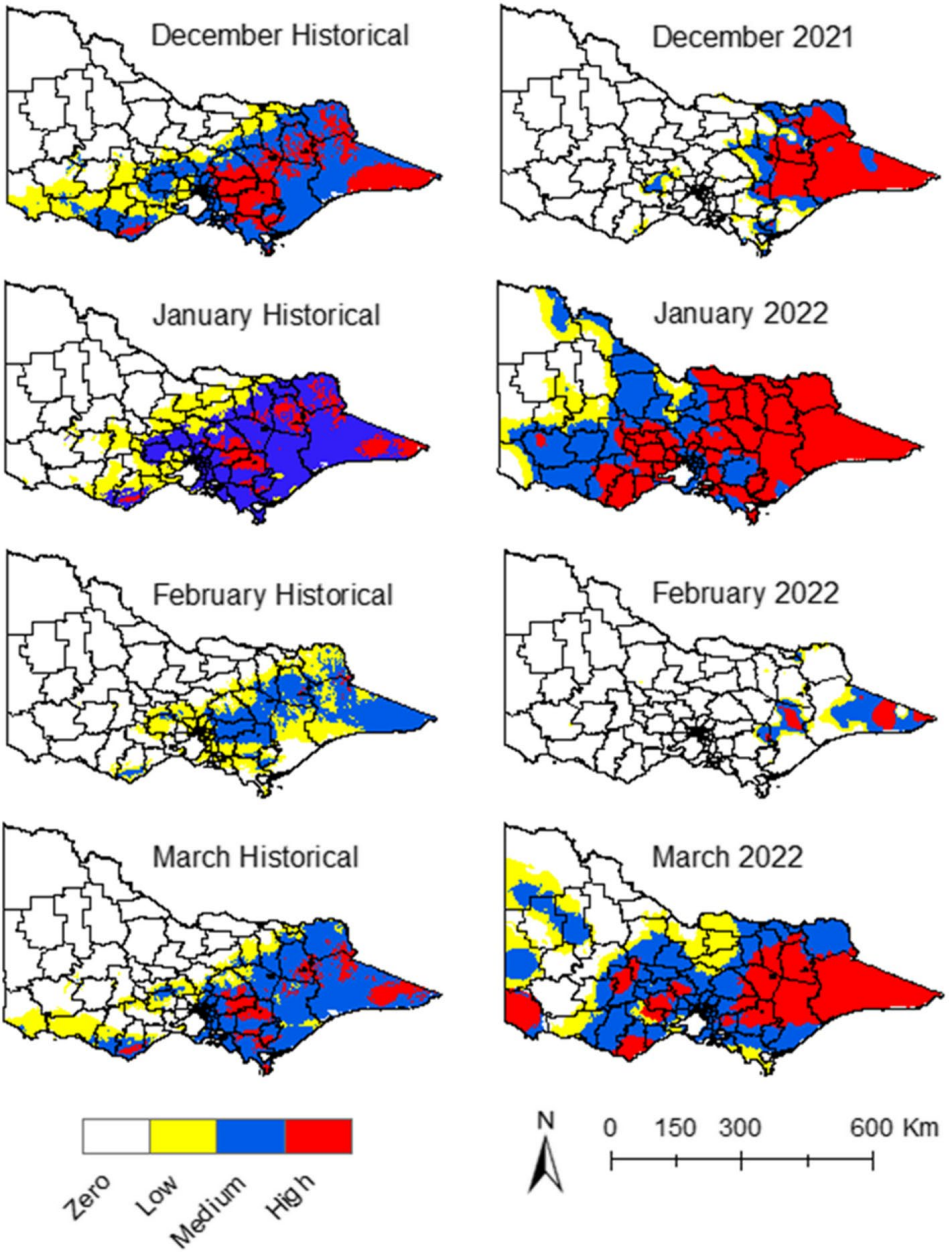
Precipitation suitability maps for JEV transmission

Historical data for precipitation showed suitability in the east, northeast, south, and southwest regions of the state during the summer season (Fig. 4). The drier north and northwest parts of the state showed low suitability for JEV transmission (Fig. 4). In January 2022, there was a discernible increase in precipitation suitability. This resulted in more LGAs presenting high suitability for JEV transmission, particularly in the east, north-east, south, and south-west regions of the state (Fig. 4). The same was observed in March, compared to the historical suitability of the same month (Fig. 4).

#### Composite climate suitability

**Fig. 5:**
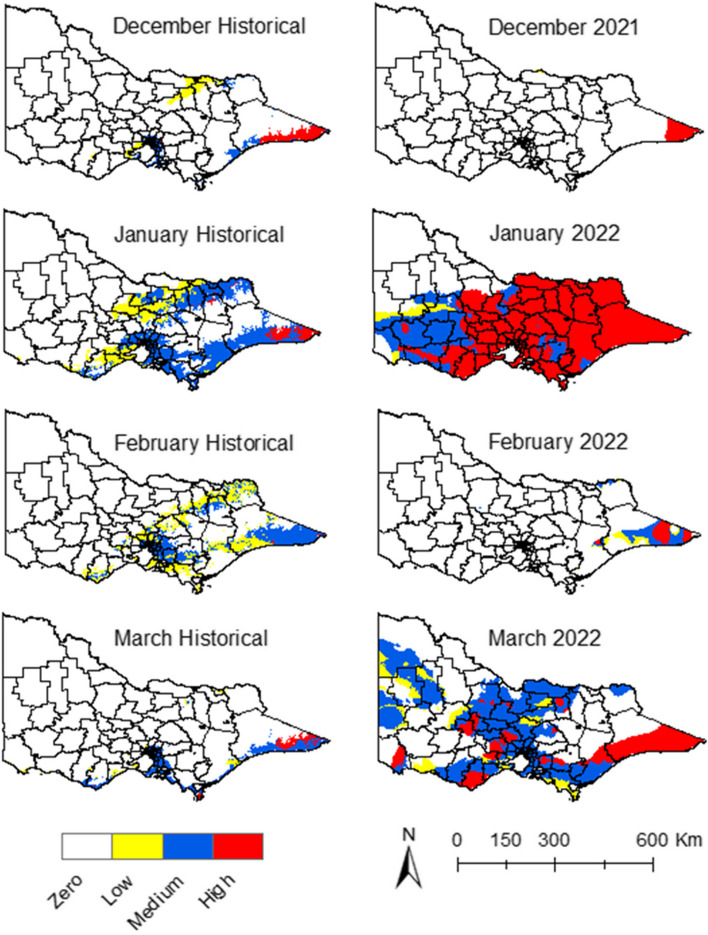
Climate suitability maps for JEV transmission

The overlay of the three key climatic factors—precipitation, maximum and minimum temperatures—resulted in the consolidated climate suitability maps (Fig. 5). With the historical climate data, some LGAs presented medium and low climate suitability for JEV in the northeast, east, and south regions of the state during January (Fig. 5). The extent of this suitability decreased during December, February, and March (Fig. 5), and with some regions around the metropolitan area with medium climate suitability throughout the season (Fig. 5). The months of January and March of 2022 had higher climate suitability compared to the same months with the historical climate data, with more LGAs presenting high and medium climate suitability (Fig. 5). In short, the precipitation and minimum temperature during January and March of 2022 had higher suitability for JEV emergence compared to historical climate data for the same months (Figs. 2, 4).

#### Risk maps

**Fig. 6:**
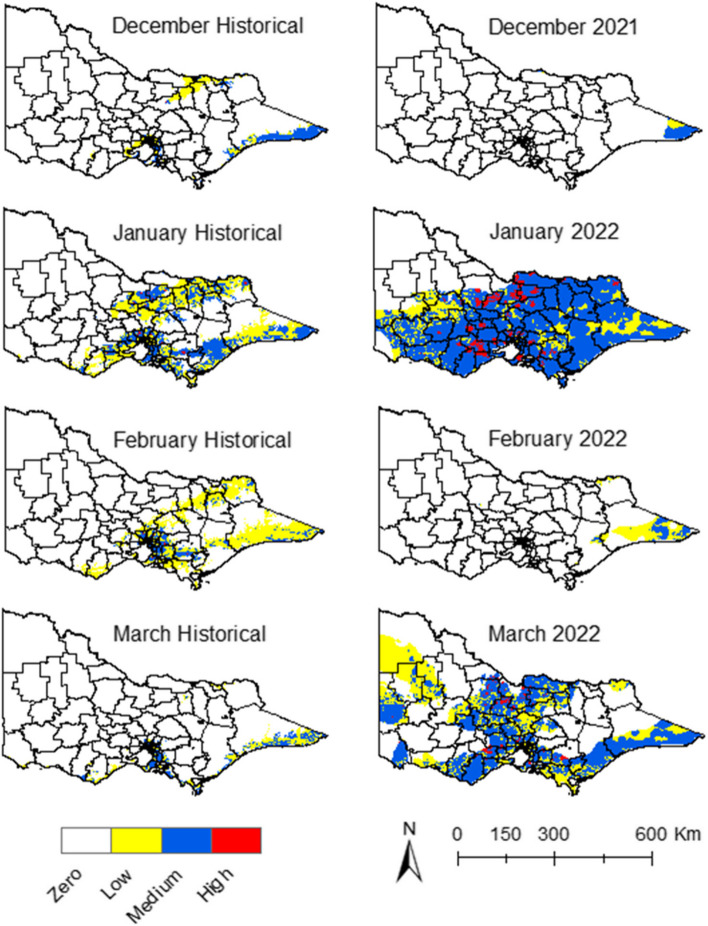
Risk maps for JEV emergence. Historical data vs. 2021–22 data

Risk maps were generated by overlaying the climate suitability data with the proximity to wetlands and piggeries, and human population density (Fig. 6). Risk maps with historical climate data for January showed that some areas in the northeast, south, and southeast of the state presented a low and medium risk (Fig. 6). The risk decreased in December, February, and March, with some regions around the metropolitan area showing a medium risk in February and March (Fig. 6).

During the summer of 2022, a larger area was exposed to the risk of JEV emergence during January and March compared to the historical risk (Fig. 6). Areas in the north, centre, and south of the state had high-risk clusters around piggeries locations during January (Fig. 6). The geographic extension and intensity of the risk of JEV emergence decreased during February and increased again in March with areas in the north, centre, south and southeast exposed to medium risk. The months of December 2021 and February 2022 had similar risks to the historical ones (Fig. 6).

**Fig. 7:**
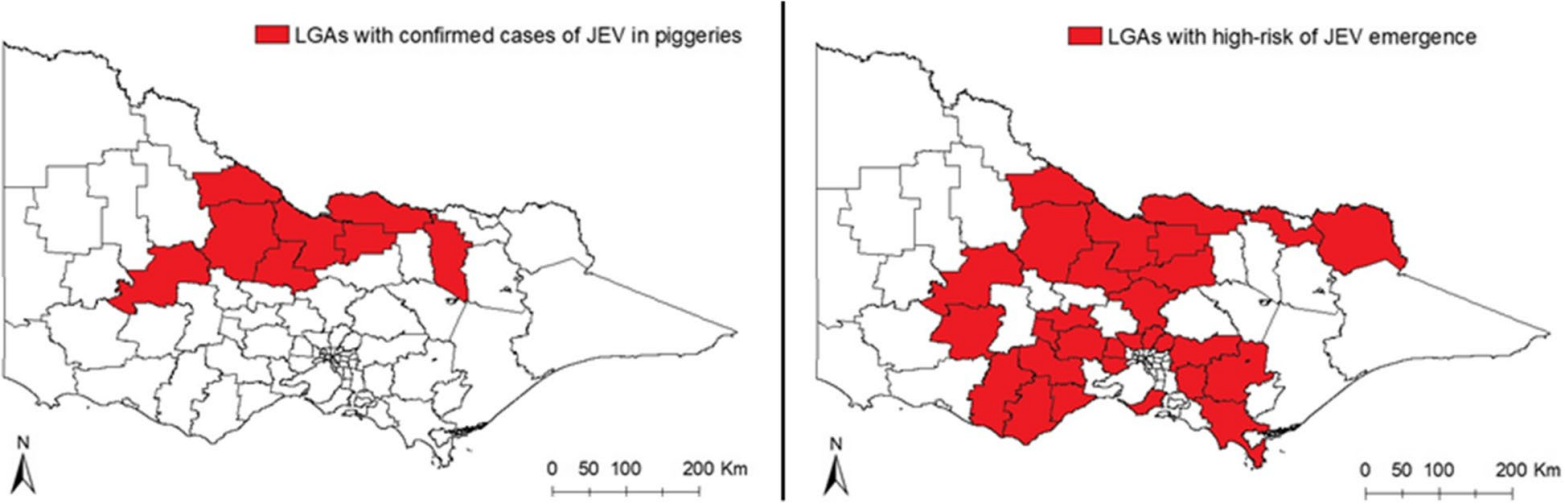
LGAs with confirmed cases of JEV cases detected in piggeries during 2021–22 vs. LGAs with a high risk of JEV emergence determined by the model during the summer season in 2021–22

Figure 7 shows the LGAs that had confirmed cases of JEV in piggeries and the LGAs that the model identified as high-risk for JEV emergence. The LGAs classified as high-risk with this model were the ones with at least one area of high risk during the period from December 2021 to March 2022. Seven out of eight of the LGAs with confirmed cases fell under the high-risk prediction.

#### Sensitivity analysis

**Fig. 8:**
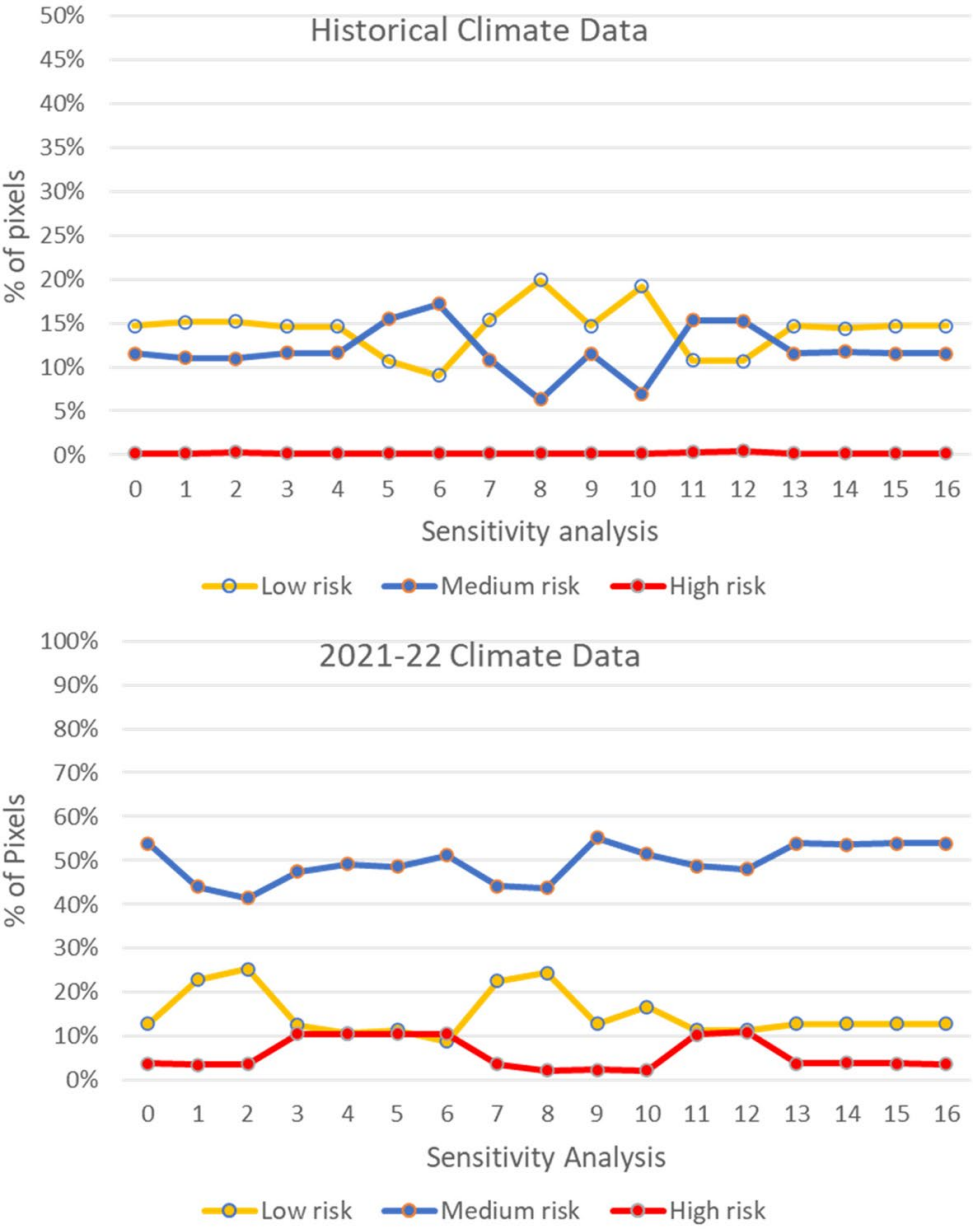
Sensitivity analysis with historical and 2022 data from January

A sensitivity analysis was conducted by changing ± 15% and ± 25% of the original weight for each criterion, while the other criteria were proportionally decreased or increased and keeping the sum of the weights equal to one (Table 1). A total of 16 sensitivity analyses were conducted for the historical and 2022 data for January. The outcomes of the sensitivity analyses were measured by counting the changes for each rating (low, medium, and high risk) in the percentage of pixels. The results of the sensitivity analyses with the historical climate data and data from 2022 (Fig. 8) presented some variation in the outcomes in the analysis when changing the weights of proximity to piggeries and wetlands (Fig. 8). However, the high-risk areas only changed in the sensitivity analysis of 2022, while the medium and low presented some changes in the historical data analysis (Fig. 8).

**Fig. 9:**
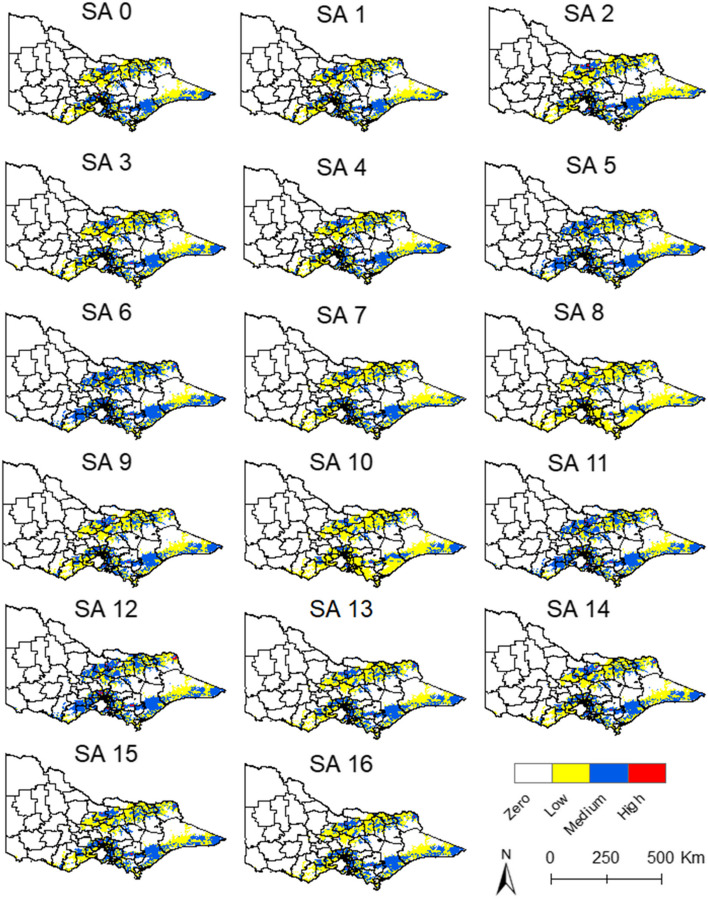
Sensitivity analysis with historical data from January. SA = sensitivity analysis

**Fig. 10:**
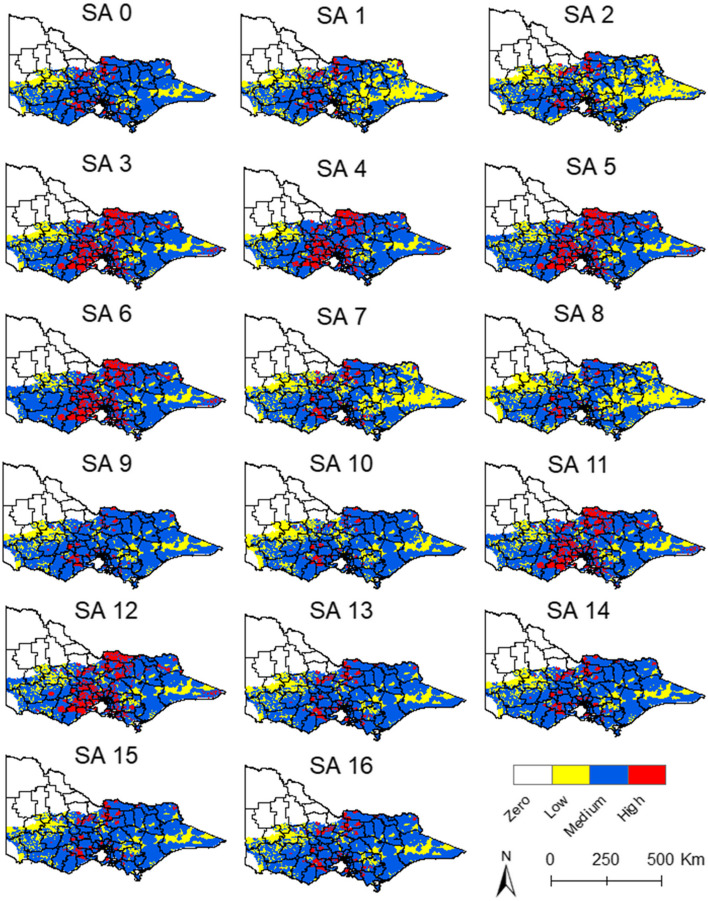
Sensitivity analysis with 2022 data from January. SA = sensitivity analysis

Figures 9 and 10 are the maps of each sensitivity analysis for the historical data and the 2022 data respectively. The maps with historical data showed that the weights of the proximity to piggeries and wetlands changed the geographic extension of the medium-risk areas, but always within the areas with suitable climate for JEV transmission. The variation in the outcomes is more evident in the 2022 maps, where the weight of the proximity to piggeries and wetlands affects the geographic extension of the high-risk areas (Fig. 10). However, the overall location of the high-risk areas in the north, central, and south regions remain constant.

## Discussion

In recent years, the modelling of VBDs has advanced, employing spatial and temporal trend analysis to forewarn of potential future vector and disease expansions, particularly within various climate change scenarios [11, 17] GIS has established itself as a vital tool in VBD modelling, facilitating the mapping of areas vulnerable to vector species proliferation and territory expansion, as delineated in various studies [53–55].

The synergistic approach of integrating GIS with an AHP analysis has demonstrated substantial efficacy in forecasting disease risk and demarcating high-risk areas [29–35]. Furthermore, GIS serves as a precise tool in risk mapping, capable of pinpointing risk areas at a micro level, hence proving indispensable in identifying vulnerable localities [53, 54].

The application of GIS goes beyond mapping; it functions as a crucial component of spatial decision support systems, aiding decision-makers in developing informed strategies, especially concerning public health preparedness against emerging infectious diseases [23, 24]. Along these lines, our study conceptualised and executed a GIS-AHP model to discern areas within Victoria, Australia, susceptible to the emergence of JEV. By assimilating historical climate data and recent 2022 data (the year marking the emergence of JEV in Australia), we aspire to contribute a robust tool in the evolving domain of public health preparedness.

The integration of expert insights into the AHP enables the formulation of a consensus regarding the influence of various criteria. Notwithstanding, some disparities were noted in the weights assigned to certain criteria by different participants (Table 2). This variance could be attributed to the diverse professional backgrounds of the participants and possibly signifies gaps in current knowledge or data paucity about VBDs. Despite these disparities, a consensus on the consolidated weight values for each criterion was achieved among the majority of participants.

Climate variables significantly influence mosquito proliferation, thereby increasing the risk of disease emergence [56]. Mosquito abundance is cumulatively influenced by both seasonality and previous weather conditions [14]. Furthermore, temperature acts as a critical determinant governing vector and pathogen survival, their geographic distribution, and the transmission dynamics of the disease [38, 57]. Nonetheless, the influence of temperature on disease transmission exhibits a non-linear pattern, as it experiences daily, monthly, and yearly fluctuations, accompanied by a lag phase between the optimal temperature and the actual disease transmission [38]. This study did not incorporate considerations of time lags and temperature fluctuations in assessing disease emergence risk, but it is postulated that a one-month time lag may be anticipated for JEV incidence [10].

Our model facilitates the extraction of individual climate condition data layers, which can subsequently be overlaid to generate a comprehensive climate suitability map (Fig. 5). This mapping process elucidates the specific conditions driving climate suitability and helps unravel the interactions of all pertinent conditions. The AHP analysis also aids in navigating the complexities associated with the amalgamation of these conditions. Notably, minimum temperature emerged as a strong predictor of JEV incidence, as supported by other studies [10, 58]. During 2022, the minimum temperature was responsible for the greatest increase in suitability, compared to historical trends. Many LGAs reported minimum temperatures exceeding the average by 3.65^0^C in January 2022 [59], creating conditions for JEV transmission during that period.

During January and March of 2022, a noticeable increase in precipitation-driven suitability was observed, with more LGAs exhibiting medium to high suitability compared to the historical data, a consequence of above-average precipitation in certain regions. An overall increase in climate suitability for JEV transmission was predominantly noted in these months, which could be attributed to a simultaneous increase in both minimum temperature and precipitation suitability. This contrasted with the maximum temperature suitability, which remained relatively consistent with historical data, indicating no significant changes that might affect JEV emergence in the 2021–22 period.

When analysing the consolidated weights assigned to different climate conditions, it becomes evident that the summer season of 2021–22 marked heightened suitability, primarily driven by increased precipitation and minimum temperature levels. These elements were recognised as pivotal factors influencing JEV transmission, as corroborated by expert consensus.

Nevertheless, it is crucial to underscore that this study was limited by the omission of certain influential factors such as geographic barriers, elevation, and anthropogenic modifications to the landscape. Consequently, the projected climate suitability areas might encompass a larger region than the actual potential habitat of the mosquito vectors [21]. Additionally, the chosen variables considered in this model may not encapsulate the complete array of factors dictating the spatial distribution of these vectors, signifying that areas identified as climatically suitable may not invariably harbor the vector population [21]. Hence, even though an area might exhibit climate suitability, it is not certain that the vector will occupy that location. It is also pertinent to mention that host availability remains a requisite determinant in assessing disease risk, an aspect only partially incorporated in this study by considering the piggeries’ location. The inclusion of other hosts population distribution and dynamics should be addressed in future research, and it represents a limitation of the current study. This project primarily focused on delineating climate prerequisites conducive to vector and virus transmission.

In assessing the potential emergence of diseases, this study integrated several risk factors such as climate variables, proximity to piggeries and wetlands, and human population density. By combining these risk factors, we managed to pinpoint regions within the state that were at a heightened risk for disease emergence. In particular, an increase in JEV incidence was observed in January and March of 2022, compared to historical data. This revealed that piggeries located in the north-central-south regions in the state were susceptible to JEV emergence, a trend significantly influenced by climate factors. It is however important to note that this does not equate to a definite presence of the disease in all piggeries within the specified regions. Instead, the model aims to identify areas potentially at risk. In this respect, evidence of the model’s predictive powers was demonstrated by accurate identifications of 7 out of 8 LGAs that reported positive cases of JEV in piggeries during the summer of 2022, representing a high sensitivity. One drawback, however, is the low specificity of the model since more LGAs were categorised as high-risk than the number of LGAs with confirmed cases during the JEV outbreak. This might be due to the lack of other hosts’ availability and dynamics data, and aspects of the disease’s ecology that is still not well understood.

The sensitivity analysis showed a pronounced correlation between high climate suitability, such as that witnessed in January of 2022, and the relative proximity to piggeries, highlighting a notable sensitivity to alterations in output data (Fig. 10). This underscores the importance of incorporating any amplifying host locations to precisely delineate areas vulnerable to increased disease emergence risks under conditions of high climate suitability. A similar trend was observed in relation to the proximity to wetlands, where a discernible sensitivity to changes was noted in scenarios exhibiting high climate suitability (Fig. 10).

For the enhancement of this model, incorporating data about the habitats of feral pigs and other potential hosts would be a prudent step. This further emphasises the necessity to incorporate climate change and climate change projections to ascertain the climate suitability for the emergence of diseases, thereby fostering a more robust and predictive approach to managing and mitigating the risks associated with infectious diseases.

Commercial piggeries are critical nodes in the propagation of JEV [60]. The vaccination of pigs presents logistical challenges as a preventative measure against JEV, given their early slaughter age of 6–8 months. Consequently, the strategic position of piggeries emerges as a viable strategy to curb JEV transmission to humans [61]. This model can provide a useful tool to aid in the planning or relocation of piggeries by delineating areas with high climate suitability and potential disease transmission risks. Given that Victoria ranks as Australia’s second-largest pork producer, with the industry witnessing rapid growth (doubling from 2018–19 to 2019–2020) [62], prudent planning for piggery locations becomes important, especially in light of the recent emergence of JEV in the state.

Incorporating both spatial and temporal analyses with an AHP enables the capture of certain complex facets that drive VBDs [14, 63]. This synthesis offers substantial value in bolstering decision-making processes aimed at enhancing preparedness and formulating adaptative strategies to combat VBDs. However, it should be noted that this model does not encompass all aspects integral to the transmission cycle of VBDs. It serves as a supportive tool in steering planning and surveillance initiatives and should ideally be utilised in conjunction with other predictive models to facilitate a more comprehensive analysis. In that way, it is possible to craft a robust predictive network that can more accurately anticipate disease spread patterns, thereby enabling proactive strategies in disease management and prevention.

In this model, facets such as the adaptation and evolution of vectors and pathogens were not considered, a potential limitation noted previously [17]. Consequently, the projected risk in some areas could potentially be higher since host and vector data were not included in the analysis. Although human population density was factored into the study, given its relevance to assessing the risk of emergence in human populations, this does not negate the potential emergence risk within host populations in the areas identified as high-risk by the model.

The benchmarks for optimal virus temperatures were derived and obtained from the previous research [38]. Due to the absence of specific data pertaining to the transmission dynamics of JEV by *Cx. annulirostris*, we approximated the transmission values utilising data from the closely related MVEV. It is important to note that there could be variations in the optimal temperature thresholds for disease transmission, and these might be subject to fluctuations based on different climatic regions [10]. Nonetheless, we anticipate these variations to exert minimal influence on the model’s overall outcome and its intended purpose. In addition, this study only considered the climate prerequisites necessary for vector transmission, potentially resulting in a biased outcome. This stems from the fact that individual species involved in the disease transmission cycle may necessitate distinct environmental conditions [17]. Moving forward, a more encompassing approach that integrates a wider spectrum of environmental variables and species-specific data can potentially facilitate a more nuanced and accurate predictive model, enhancing our preparedness and response strategies in managing VBDs.

## Conclusions

The results obtained from the GIS-AHP-based model developed in this research successfully delineated areas with elevated risk of JEV emergence during the summer of 2022, highlighting the central role of climate change in escalating the risk associated with the disease. This underscores the potential utility of this model as a first step to support the prediction of the emergence and patterns of other VBDs by integrating pertinent risk factors and climate projections into the analysis framework. Further work is needed to ensure more specific predictions by incorporating more variables into the analysis.

By employing this model, a significant stride is made in enhancing the decision-making capabilities of various stakeholders including policymakers, public health authorities, land-use planners, and academic researchers focused on VBDs. Furthermore, the model can serve as a starting point to facilitate more comprehensive local risk assessments, and consequently, fortifying public health preparedness strategies against VBDs.

Looking forward, refining this model to encompass a broader spectrum of variables such as geographic barriers, elevation, human modifications to the landscape, and the environmental requirements of each species involved in the disease cycle, would pave the way for more nuanced and accurate risk predictions. Moreover, incorporating data on host and vector distributions can further augment the predictive capacity of this model.

To summarise, this model signifies a first yet promising step in developing a resource aimed at streamlining localised risk assessments and fostering an environment of proactive response and preparedness against emerging threats of VBDs under climate change. Its adaptable nature aligns well with the evolving landscape of vector-borne disease epidemiology, offering a foundation structure that can be modified and enhanced to specific diseases and locations as required.

## Data Availability

The datasets used and/or analysed during the current study are available from the corresponding author on reasonable request.
